# Characterization of Glial Cell Models and *In Vitro* Manipulation of the Neuregulin1/ErbB System

**DOI:** 10.1155/2014/310215

**Published:** 2014-08-07

**Authors:** Davide Pascal, Alessia Giovannelli, Sara Gnavi, Stefan Adriaan Hoyng, Fred de Winter, Michela Morano, Federica Fregnan, Paola Dell'Albani, Damiano Zaccheo, Isabelle Perroteau, Rosalia Pellitteri, Giovanna Gambarotta

**Affiliations:** ^1^Department of Clinical and Biological Sciences, University of Torino, Nerve Regeneration Group, 10043 Orbassano, Italy; ^2^Neuroscience Institute Cavalieri Ottolenghi (NICO), 10043 Orbassano, Italy; ^3^Department of Neuroregeneration, Netherlands Institute for Neuroscience, Royal Netherlands Academy of Arts and Sciences (KNAW), 1105 BA Amsterdam, The Netherlands; ^4^Department of Neurosurgery, Leiden University Medical Center, 2333 ZA Leiden, The Netherlands; ^5^Institute of Neurological Sciences, National Research Council (CNR), 95126 Catania, Italy; ^6^Department of Experimental Medicine, Section of Anatomy, University of Genova, 16132 Genova, Italy; ^7^Neuroscience Institute of Torino (NIT), Interdepartmental Centre of Advanced Studies in Neuroscience, University of Torino, 10043 Orbassano, Italy

## Abstract

The neuregulin1/ErbB system plays an important role in Schwann cell behavior both in normal and pathological conditions. Upon investigation of the expression of the neuregulin1/ErbB system *in vitro*, we explored the possibility to manipulate the system in order to increase the migration of Schwann cells, that play a fundamental role in the peripheral nerve regeneration. Comparison of primary cells and stable cell lines shows that both primary olfactory bulb ensheathing cells and a corresponding cell line express ErbB1-ErbB2 and neuregulin1, and that both primary Schwann cells and a corresponding cell line express ErbB2-ErbB3, while only primary Schwann cells express neuregulin1. To interfere with the neuregulin1/ErbB system, the soluble extracellular domain of the neuregulin1 receptor ErbB4 (ecto-ErbB4) was expressed *in vitro* in the neuregulin1 expressing cell line, and an unexpected increase in cell motility was observed. *In vitro* experiments suggest that the back signaling mediated by the transmembrane neuregulin1 plays a role in the migratory activity induced by ecto-ErbB4. These results indicate that ecto-ErbB4 could be used *in vivo* as a tool to manipulate the neuregulin1/ErbB system.

## 1. Introduction

The neuregulin1/ErbB system plays an important role in Schwann cell (SC) behavior both in normal and pathological conditions [1] and the possibility to manipulate it gives new perspectives to improve posttraumatic nerve regeneration [2–4].

The ErbB receptor family consists of four tyrosine kinase receptors: epidermal growth factor receptor (EGFR, also called ErbB1 or HER1), ErbB2, ErbB3, and ErbB4 [5]. ErbB receptors bind several structurally related growth factors. Among them, neuregulin1 (NRG1) is the most characterized and studied in the peripheral nervous system (PNS) for its role in axon-glial signaling and SC activity. The NRG1 gene codes for more than 20 different isoforms [1, 6–8] which differ because of alternatively spliced exons. Actually, soluble and transmembrane isoforms were described that differ in the presence of N-terminal domains and their signaling mode: soluble isoforms (types I and II) are mainly released by glial cells and signal in a paracrine/autocrine manner; transmembrane isoforms (type III) are mainly expressed by axons and signal in a juxtacrine manner [8]. NRG1 are further classified as alpha and beta isoforms, according to the characteristics of their EGF-like domain.

It has been shown that transmembrane ligand-receptor interactions may lead to a process of back-signaling, which is mediated by the action of a *γ*-secretase enzyme which causes the release of a cytoplasmic fragment able to translocate into the nucleus [9, 10].

In order to better study the role of the NRG1/ErbB system in the peripheral nerve, four different* in vitro* models were analyzed: primary rat SC harvested from sciatic nerve, a SC line (RT4-D6P2T) [11], primary glial cells of the olfactory nerve, known as olfactory ensheathing cells (OEC), and a neonatal olfactory bulb ensheathing cell line (NOBEC) [12].* In vitro* experiments were carried out to address these questions.

 Are NRG1 and ErbB receptor expressed in these four cellular models? What are the* in vitro* effects of manipulating the NRG1/ErbB system by expression of the soluble extracellular domain of the NRG1 receptor ErbB4 (ecto-ErbB4) in glial cells?

## 2. Materials and Methods

### 2.1. *In Vitro* Assays

#### 2.1.1. Cultures of NOBEC, RT4-D6P2T, and COS7

Neonatal olfactory bulb ensheathing cells (NOBEC) line, derived from primary cells dissociated from neonatal rat olfactory bulb and immortalized by retroviral transduction of SV40 large T antigen [12], was kindly provided by Dr. Jacobberger (Comprehensive Cancer Center, Case Western Reserve University, 10900 Euclid Avenue, Cleveland, OH 4106-4944, USA). Rat RT4-D6P2T [11] and COS7 were provided by the American Type Culture Collection (ATCC). Cell lines were grown as monolayer at 37°C in a humidified atmosphere of 5% CO_2_/air, in Dulbecco's modified Eagle's medium (DMEM, Invitrogen, UK) supplemented with 100 U/mL penicillin, 100 *μ*g/mL streptomycin, 1 mM sodium pyruvate, 2 mM l-glutamine, and 10% heat-inactivated foetal bovine serum (FBS; Invitrogen). Recombinant NRG1*β*1 has been produced in the laboratory as a His-tag fusion protein in* E. coli *[13].

#### 2.1.2. Cultures of Primary Schwann Cells (SC)

To harvest Schwann cells (SC), rat sciatic nerves were exposed, removed, and kept in DMEM plus glutamax (Invitrogen, UK) containing 100 U/mL penicillin and 100 *μ*g/mL streptomycin. Nerves were then dissected in trunks, desheathed, and finally chopped in 1 mm segments. The segments were then plated in a Petri dish in SC growth medium (DMEM plus glutamax containing 100 U/mL penicillin, 100 *μ*g/mL streptomycin, 14 *μ*M forskolin, and 100 ng/mL NRG1*β*1, R&D Systems, UK). Cells were incubated for 2 weeks at 37°C with fresh medium added approximately every 72 h. After these 2 weeks medium was aspirated and 0.125% (w/v) collagenase type IV and 117 U/mg dispase were added to the Petri dish. After 24 hours (h) incubation, cell suspension was filtered through a 70 mm cell strainer (Falcon; BD Biosciences Discovery Labware, Bedford, MA) and centrifuged at 100 ×g for 5 min to obtain the cell pellet. Finally, the cell pellet was resuspended in SC growth medium, seeded into a Petri dish pre-coated with poly-D-lysine (Sigma, St Louis, MO, USA), and incubated in the same conditions. The following day, the medium was changed and cells were left to proliferate. When confluent, SC were purified by an antibody complement method to eradicate the remaining fibroblasts [14–16].

#### 2.1.3. Cultures of Primary Olfactory Ensheathing Cells (OEC)

OEC were isolated from 2-day-old rat pups (P2) olfactory bulbs using an already described method [17]. Ten neonatal rats were used to produce each batch of OEC. Initial steps involved peeling away the olfactory nerve layer from the rest of the bulb and digesting the tissue in MEM-H containing 0.03% collagenase and 0.25% trypsin for 15 min at 37°C. This step was repeated twice with a fresh solution. Trypsinization was stopped by adding 10% foetal calf serum (FCS)-DMEM. The digested tissue was mechanically dissociated by trituration and filtrated through a 80 *μ*m nylon mesh followed by centrifugation at 500 ×g for 10 min. Cells were resuspended and plated in flasks, fed with fresh 10% FCS-DMEM, andsupplemented with 2 mM L-glutamine, 50 U/mL penicillin, and 50 *μ*g/mL streptomycin. 24 h after initial plating, 10 *μ*M antimitotic agent cytosine arabinoside was added to reduce the number of dividing fibroblasts. OEC cultures were further processed by passing cells from one flask to another. This step reduces contaminating cells because they adhere more readily to plastic than OEC. In the last passage OEC were plated on 25 cm^2^ flasks and cultured in 10% FCS-DMEM supplemented with bovine pituitary extract. OEC purity was verified using immunocytochemistry with p75 and S-100. The percentage of S-100/p75 positive cells in the cultures was ~85–90% (data not shown). Cells were incubated at 37°C in 10% FCS-DMEM and the medium was changed twice a week.

#### 2.1.4. GDNF Stimulation Assay

Purified primary OEC and NOBEC were plated on 14 mm diameter poly-L-lysine (PLL, 10 *μ*g/mL, Sigma) coated glass coverslips at a final density of 3 × 10^3^ cells/coverslip and grown both in 10% FCS-DMEM and in serum-free DMEM. Cells were cultured with the addition of Glial Derived Neurotrophic Factor (GDNF, 1 ng/mL, Chemicon) for eight days, changing the medium twice. Control cultures received medium with no addition of trophic factor (CTR). Then, cells were processed by immunocytochemical procedures and total RNA extraction.

#### 2.1.5. Immunocytochemistry

OEC and NOBEC were fixed in 0.1 M phosphate buffer pH 7.4 (PBS) containing 4% paraformaldehyde (PAF) for 30 minutes (min). After washing in PBS cells were treated with PBS containing 5% normal goat serum (NGS), 0.1% Triton X-100 at room temperature (RT) for 15 min. OEC and NOBEC were incubated overnight at 4°C with the following primary antibodies: anti-S-100 (mouse, working dilution/w.d. 1 : 100; Sigma Aldrich), antinestin (rabbit, w.d. 1 : 100; Immunological Science), and antivimentin (mouse, w.d. 1 : 50; Dako). After washing, cells were incubated for 45 min at RT with the correspondent anti-mouse and anti-rabbit fluorescent secondary antibodies to visualize primary antibodies. The immunostained coverslips were analyzed on a Zeiss fluorescence microscope and images were captured with an Axiovision Imaging System. No staining of cells was observed in control incubations in which the primary antibodies were omitted.

#### 2.1.6. Cell Transfection

For transient transfection of plasmidic DNA, NOBEC, RT4-D6P2T, and COS7 cells were transfected with Lipofectamine 2000, according to manufacturer's instructions. Efficient expression of the recombinant protein was assessed by Western blot analysis.

#### 2.1.7. Cell Migration Assay

Transwell assays were performed 48 h after DNA transfection as previously described [18]. To inhibit the *γ*-secretase, cells were pretreated with 100 *μ*M DAPT (*γ*-secretase inhibitor compound IX, Calbiochem) for 3 days. Since DAPT was diluted with DMSO, the control was carried out by treating cells with the same volume of this solvent.

For each Transwell four images were analyzed and the amount of migrated cells was evaluated as the total area of migration (in pixel^2^) was calculated with the Image J software and expressed as percentage of the total number of migrated cells for each single experiment. Cells were discriminated by the pores of the Transwell membrane by applying a threshold of 300 pixel^2^. For each experimental condition a technical triplicate was made and each experiment was repeated at least 3 times.

#### 2.1.8. RNA Isolation and cDNA Preparation

Total RNA was extracted with TRIzol (Invitrogen) according to the manufacturer's instructions, adding 5 *μ*g glycogen as a carrier to facilitate RNA precipitation. 1 *μ*g total RNA was subjected to a reverse transcriptase (RT) reaction in 25 *μ*L reaction volume containing 1X RT-Buffer (Fermentas); 0.1 *μ*g/*μ*L bovine serum albumin (BSA, Sigma); 0.05% Triton X-100; 1 mM dNTPs; 7.5 *μ*M random exanucleotide primers (Fermentas); 1 U/*μ*L RIBOlock (Fermentas); and 200 U RevertAid M-MuLV reverse transcriptase (Fermentas). The reaction was performed for 10 min at 25°C, 90 min at 42°C, and 10 min at 90°C. Control reactions “RT-” (without the enzyme RT) and “H_2_O”, without RNA, were carried out.

#### 2.1.9. Quantitative Real-Time PCR (qRT-PCR) Analysis

Quantitative real-time PCR analysis was performed using Sybr Green chemistry; data were analyzed by ΔΔCt relative quantification method normalizing to the housekeeping gene Tata-box Binding Protein (TBP) (forward primer: 5′-TAAGGCTGGAAGGCCTTGTG-3′; reverse primer: 5′-TCCAGGAAATAATTCTGGCTCATAG-3′). Real-time PCR reactions were performed using the 7300 real-time PCR system (Life Technology). Each sample was run in triplicate on 96-well optical PCR plates (Life Technology). In each well a PCR reaction was carried out on 5 *μ*L cDNA corresponding to 20 ng starting RNA (1 *μ*g RNA retrotranscribed in 25 *μ*L, diluted 1 : 10 in water), Sybr Green PCR Master Mix (Life Technology) and 300 nM primers (Life Technology) in a reaction volume of 25 *μ*L. Specific primers designed to amplify ErbB1, ErbB2, ErbB3, ErbB4, NRG1, p75NGFR, GFAP, and S100 are listed in Table 1. After an initial denaturation step for 10 min at 95°C, denaturation in the subsequent 40 cycles was performed for 15 seconds (sec) at 95°C followed by primer annealing and elongation at 60°C for 1 min. Relative amount of mRNA that had been retrotranscripted into cDNA was calculated by comparative (ΔΔCt) method. In the first step of the method, the difference between Ct values of target and housekeeping gene was calculated (ΔCt), whereas in the second step the difference between the ΔCt values of the samples and the calibrator was determined (ΔΔCt). For each gene, the cells with the highest level of expression were chosen as calibrator. The normalized relative quantity (NRQ) was determined using the formula: NRQ = 2^−(ΔΔCt)^. Results were expressed as mean + SEM.

#### 2.1.10. Total Protein Extraction and Western Blot Analysis

Total proteins were extracted by solubilizing cells in boiling Laemmli buffer (2.5% SDS, 0.125 M Tris-HCl pH6.8), followed by 3 min at 100°C. Protein concentration was determined by the BCA method, and equal amounts of proteins (denaturated at 100°C in 240 mM 2-mercaptoethanol and 18% glycerol) were loaded onto each lane, separated by SDS-PAGE, transferred to a HybondTM C Extra membrane as previously described [19]. Primary antibodies used are ErbB1 (#sc-03), ErbB2 (#sc-284), ErbB3 (#sc-285), ErbB4 (#sc-283), NRG1 (#sc-347, #sc-348), w.d. 1 : 1000, from Santa Cruz; p-AKT (#4051), AKT (#9272), p-ERK (#9106), ERK (#9102) w.d. 1 : 1000, from Cell Signaling; flag (#F7425) w.d. 1 : 10000, from Sigma; p75-NGFR (#ab52987), w.d. 1 : 2000, from Abcam; GAPDH (#4300), w.d. 1 : 20000, and from Ambion; secondary antibodies used are horseradish peroxidase-linked anti-rabbit (#NA934) and anti-mouse (#NA931) w.d. 1 : 40000, from GE Health.

#### 2.1.11. Nuclear and Cytoplasmic Protein Extraction

Cell plates were washed 3 times with PBS on ice. On each plate, 300 *μ*L of lysis buffer (20 mM Tris-HCl pH8.0, 20 mM NaCl, 0.5% NP40, plus an anti-protease cocktail, Roche) was added for 10 min at 4°C.

After lysis, cells and nuclei were collected and centrifuged for 30 min at 3000 rpm at 4°C, in order to separate the nuclear and cytoplasmic material; the supernatant, which contains the cytoplasmic extract, was separated from the pellet, syringed with a G26 needle and then centrifuged for 5 min at 12000 rpm at 4°C. Supernatant was collected, aliquoted, and frozen at −80°C. The pellet obtained after the first centrifuge, containing nuclear material, was washed 3 times with lysis buffer and centrifuged, in order to remove cytoplasmic protein traces. Pellet was resuspended in 30 *μ*L buffer C (20 mM Hepes pH8.0; 1.42 mM NaCl, 1.5 mM MgCl_2_, 1.2 mM EDTA); then, 30 *μ*L Buffer C containing 50% glycerol was added, and nuclear proteins were incubated on ice for 30 min. The treatment with this hyperosmotic buffer causes cell nucleus collapse and nuclear protein spillage. After incubation, extracts were centrifuged for 90 sec at 12000 rpm at 4°C. The supernatant containing nuclear proteins was collected, centrifuged again, diluted 1 : 3 in 20 mM Hepes pH 8.0, and stored at −20°C.

### 2.2. Construct Preparation

#### 2.2.1. Cloning Strategies for NRG1-ICD-NLS and NRG1-ICD-ΔNLS Constructs

Because the C terminal cytoplasmic domain is common to all NRG1 isoforms, the cDNA coding for NRG1-typeI-*β*1a (kindly provided by K. Lay, accession number NM_013956) was used as template to clone the cytoplasmic domain of NRG1. To allow expression of the protein, an artificial start codon (ATG, shown in bold in the primer sequence) was added on the forward primer, inserted in a Kozac sequence. To obtain an intracellular domain (ICD) containing the nuclear localization signal (NLS) the following primers were used:

 forward: 5′-TAGCCTGCAGC**ATG**GGCAAAACCAAGAAACAGCG-3′;
 reverse: 5′-ATCGATATCTACAGCAATAGGGTCTTGGTTAG.

To obtain an intracellular domain (ICD) missing the nuclear localization signal (ΔNLS), the following primers were used:

 forward: 5′-TAGCCTGCAGC**ATG**GAGCTTCATGATCGGCTCC-3′;
 reverse: 5′-ATCGATATCTACAGCAATAGGGTCTTGGTTAG.

Restriction enzymes sequences (underlined) were added to the primers to facilitate the subcloning (PstI in the forward primer, EcoRV in the reverse primer).

Amplification reactions were carried out using 0,8 ng template (NRG1-tipoIII-*β*1a) and the AmpliTaq Gold enzyme, following manufacturer's instructions, in the Thermal Cycler GeneAmp PCR System 2400 (Perkin Elmer). The amplification was performed according to the following protocol: 5 min at 94°C; then 40 cycles: 30 sec at 94°C, 30 sec at 60°C, 90 sec at 72°C; and 20 min at 72°C. Amplification products were cloned into the pGEM-T vector, using chemically competent JM-109 cells (Promega), following manufacturer's instructions. Two clones, corresponding to the constructs ICD-NLS and ICD-ΔNLS, were completely sequenced (BMR Genomics Laboratories). Inserts were recovered by EcoRV and NcoI digestions, sticky ends were blunted and inserted into pIRESpuro2 (Clontech) previously cut with NotI, blunted and dephosphorylated.

#### 2.2.2. Cloning Strategies for Ecto-ErbB4-FLAG Construct and Subcloning into Lentiviral Vector

To obtain a construct to express the extracellular domain of ErbB4 (ecto-ErbB4) fused with a FLAG epitope, the ErbB4 extracellular domain, recovered from the pIRES-puro2-ErbB4-JMa-cyt2 construct [18] by EcoRV and BbsI digestion and blunting, was subcloned into the EcoRV site of the pCMV-Tag4a vector (Stratagene). To obtain a lentiviral vector to express ecto-ErbB4-FLAG, the insert with the FLAG and the following STOP codon was recovered from pCMV-Tag4c vector by EcoRV and KpnI digestion and blunting and subcloned into the multiple cloning sites (mcs) of the lentiviral vector pRRL-CMV-mcs-WPRE, flanked by the constitutively active CMV promoter and the woodchuck hepatitis virus posttranscriptional regulatory element (WPRE).

### 2.3. Lentivirus Production and RT4-D6P2T Infection

LV stocks were generated as previously described [20–22]. Briefly, two 15 cm diameter Petri dishes containing 1.25 × 10^7^ HEK293T seeded in Iscoves modified Dulbecco's medium (IMDM) containing 10% FCS, 100 U/mL penicillin, 100 *μ*g/mL streptomycin (PS), and Glutamax (Invitrogen) were prepared. Using branched polyethylenimine (Sigma, St Louis, MO) a triple transfection with the LV transfer, packaging (pCMVdeltaR8.74) and envelope (pMD.G.2) plasmid, was performed (ratio 3 : 2 : 1, total DNA 90 *μ*g/plate). After 14 h, the medium was replaced by IMDM containing 2% FCS, 1% PS and Glutamax. After 24 h, the medium was harvested, filtered through a 0.22 *μ*m filter, and concentrated by ultracentrifugation at 20.000 rpm for 2.5 h in a SW32Ti rotor (Beckman Coulter B, The Netherlands). Viral pellets were resuspended in PBS pH 7.4 aliquoted and stored at −80°C until further use. Serial dilutions (10^−2,−3,−4^ and 10^−2  to  −7^ for LV-GFP) of all viral stocks were used to infect 2 × 10^5^ HEK293T in IMDM 2% FCS, 1% P/S and Glutamax seeded in poly-L-lysine (PLL) coated 24-well culture plates. After 48 h the number of transducing units per mL (TU/mL) for the LV-GFP stock was manually quantified by counting transduction events in the LV-GFP transduced cells using a fluorescence microscope (10^−5,−6,−7^) and genomic DNA (gDNA) of all samples was extracted and measured for viral integrating events by quantitative PCR (10^−2,−3,−4^). Briefly, cells were harvested and gDNA was extracted (DNeasy Blood & Tissue Kit, Qiagen, Venlo, the Netherlands). Viral mediated transgene integration was measured using primers directed against the lentiviral WPRE on an ABI 7900HT detection system (Applied Biosystems) using the SYBR green PCR kit (Applied Biosystems). WPRE primers sequences were as follows: 5′-TTCCCGTATGGCTTTCATTT-3′ and 5′-GAGACAGCAACCAGGATTTA-3′. All expression values were normalized to that of the reference gene GAPDH. GAPDH primers were as follows: 5′-TGCACCACCAACTGCTTAGC-3′ and 5′-CGCATGGACTGTGGTCATGA-3′. The ratio between the TU/mL and gDNA WPRE content of the LV-GFP stock was used to calculate relative TU/mL titers for all stocks on the basis of their gDNA WPRE content.

RT4-D6P2T cells seeded at 1 × 10^3^ cells/well in a 96-well plate were infected using LV-sGFP or a combination of LV-sGFP and LV-Ecto-ErbB4 FLAG. Cells were infected three times at a MOI of 100. Confluent cells were harvested, expanded, and frozen.

Western blot and immunocytochemistry were performed on confluent cells in order to evaluate virus infection efficiency and sGFP and ecto-ErbB4-FLAG expression.

Confluent cells were fixed by incubation with 4% PFA and permeabilized with 0.2% Triton X-100 diluted in PBS by 45 min incubation at RT. Blocking solution containing 5% FCS diluted in 0.2% Triton X-100 PBS was applied for 1 h at RT. Monoclonal anti-GFP antibody (1 : 500 in blocking solution, Abcam) and rabbit anti-FLAG primary antibody (1 : 500 in blocking solution, Sigma) were incubated overnight (o/n) at 4°C. Following 3 washes of 15 min each goat-anti-rabbit IgG (H+L) Cy3 and goat-anti-mouse IgG (H+L) Alexa 488 secondary antibody (Invitrogen, diluted 1 : 200 in PBS) were incubated for 2 h at RT. Following 3 washes of 15 min nuclei were stained using Hoechst (Sigma) diluted 1 : 1000 in PBS. Fluorescent images were acquired using an inverted optical microscope (Axiovert 200, Zeiss). Western blot analysis was performed as described above.

### 2.4. Ecto-ErbB4-FLAG Protein Purification

#### 2.4.1. Co-Immunoprecipitation Assay

Ecto-ErbB4-FLAG protein for coimmunoprecipitation assay was obtained from conditioned medium of COS7 transiently expressing the protein. After three days of culture, medium was collected, centrifuged and 1 mL aliquots were incubated with either 100 ng or 200 ng recombinant NRG1*β*1-Hys protein and immunoprecipitated as previously described [18] using an anti-FLAG polyclonal antibody (SIGMA #F7425). Immunoprecipitated proteins were analyzed by western blot, using an antibody directed to the hystidine tag.

#### 2.4.2. Ecto-ErbB4-FLAG Protein Production and Purification

Ecto-ErbB4-FLAG protein for* in vitro* experiments was purified from conditioned medium of RT4-D6P2T stably expressing the protein. After four days of culture in the presence of serum free medium, medium was collected, centrifuged, and filtered (0.22 *μ*m filter). Target protein was then purified using ANTI-FLAG M2 affinity gel (SIGMA #A2220) and eluted by competitive elution using 100 *μ*g/mL FLAG peptide. Eluted solution was collected in 10 fractions and a Western blot was performed to confirm the presence of target protein. Only positive fractions were frozen in liquid nitrogen, adding 15% glycerol to prevent protein damage, and stored at −80°C.

### 2.5. Statistical Analysis

Quantitative data are presented as mean + SEM. All data were statistically analyzed using *t*-test or one-way analysis of variance and post-hoc analysis by the Bonferroni test (SPSS software).

## 3. Results

### 3.1. Comparative Characterization of Primary Glial Cell Cultures and Glial Cell Lines

#### 3.1.1. Cellular Differentiation of OEC and NOBEC upon GDNF Treatment under Different Growth Conditions

**Figure 1:**
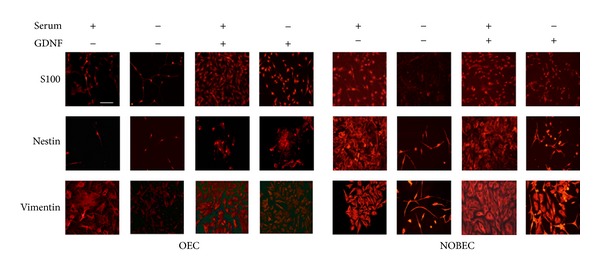
OEC and NOBEC respond similarly to GDNF stimulation. Figure shows representative fields of OEC and NOBEC immuno-stained with anti-S-100, antinestin, and antivimentin antibodies. Cells were grown for eight days after plating with and without serum and GDNF. Fields were chosen to clearly to show both the morphological aspect and the specific marker expression. Scale bars 50 *μ*m.

To characterize these cell models* in vitro*, a stimulation assay was carried out with Glial cell-derived neurotrophic factor (GDNF), a well-recognized growth factor for glial cells [23], to both primary OEC and NOBEC cell line cultures in order to evaluate its effect on cell survival and expression of glial differentiation markers. Concentration and exposure time of the cultures to the GDNF were previously established [24]. In the presence of serum, the majority of OEC and NOBEC exhibited both star and spindle shapes which are their* in vitro* typical morphological features (Figure 1).

Results show a different intensity level in the expression of some glial markers both in OEC and in NOBEC, grown in different conditions and treated with GDNF; S-100 immunoreactivity was higher in OEC and NOBEC grown in the presence of serum in comparison with cells grown in serum free medium (SFM); when GDNF was added to cultures in the presence of serum, an increased number of positive S-100 both in OEC and in NOBEC was observed.

Nestin immunoreactivity showed a more intense expression in NOBEC compared with OEC in all culture conditions and the addition of GDNF determined an upregulation of nestin both in serum and in SFM in NOBEC and OEC. Vimentin was more expressed in OEC grown in the presence of serum and GDNF addition increased the expression of vimentin in OEC grown both in serum and in SFM. NOBEC showed a major expression of vimentin compared with OEC, especially when they were grown with GDNF both in serum and in SFM (Figure 1). This different expression suggests that NOBEC cell line is more resistant to stress than OEC primary cultures and that NOBEC might represent a good glial cell model for* in vitro* assays.

#### 3.1.2. Expression Analysis of NRG1/ErbB System and Glial Markers

**Figure 2:**
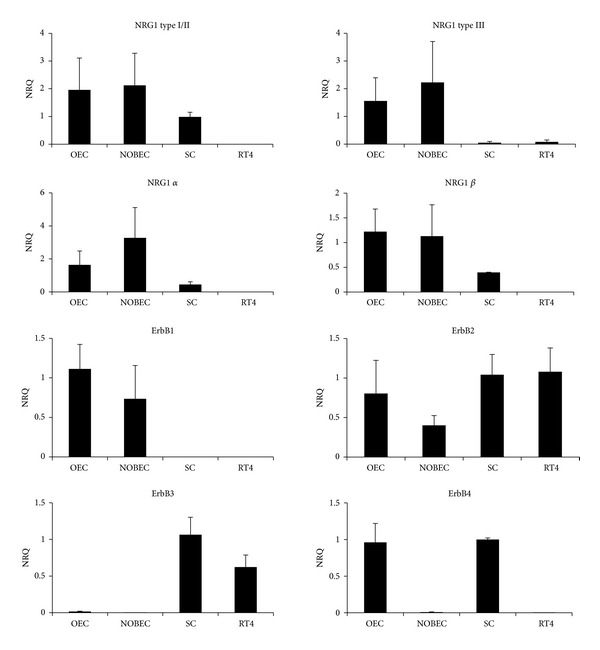
Primary cultures and cell lines derived from OEC and SC express different levels of NRG1 isoforms and ErbB receptors. Graphs show normalized relative quantification (NRQ) of the different NRG1 isoforms and ErbB receptors obtained by qRT-PCR. For each gene, the cells with the highest level of expression were chosen as calibrator (NRQ = 1). Data are presented as mean + SEM.

**Figure 3:**
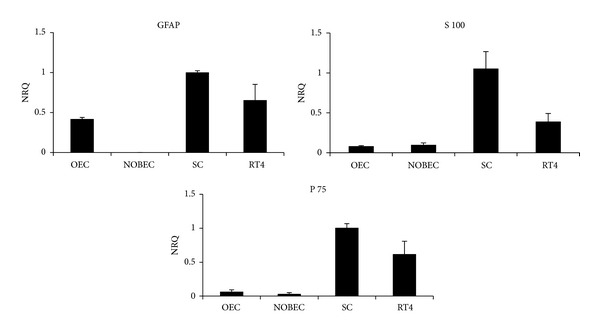
Primary cultures and cell lines derived from OEC and SC express different levels of glial genes. Graphs show normalized relative quantification (NRQ) of GFAP, S100, and p75 obtained by qRT-PCR. For each gene, the cells with the highest level of expression were chosen as calibrator (NRQ = 1). Data are presented as mean + SEM.

The mRNA expression level of the NRG1/ErbB system (Figure 2) and of glial markers (Figure 3) was examined by quantitative real-time PCR (qRT-PCR) in primary cultures of Schwann cells (SC) and olfactory bulb ensheathing cells (OEC) and was compared with the corresponding stable cell lines RT4-D6P2T (derived from a Schwannoma) and NOBEC (neonatal olfactory bulb ensheathing cells).

For each gene, the normalized relative quantity (NRQ) was determined using the sample with the highest level of expression as calibrator (NRQ = 1); therefore, the relative (and not absolute) gene expression shown in the graphs cannot be compared among different genes. Results show that ErbB1 is expressed by OEC and NOBEC, while SC and RT4-D6P2T do not express this receptor; ErbB2 is expressed by all cell types; ErbB3 is expressed by SC and RT4-D6P2T cells, barely detectable in OEC and NOBEC; ErbB4 mRNA is barely detectable only in SC and OEC.

To investigate the presence of mRNA coding for NRG1, different primer pairs were used that allow the amplification of different NRG1 isoforms [25]. Results show that RT4-D6P2T cells do not express any NRG1 isoform, whereas OEC and NOBEC express different NRG1 isoforms (type I/II, *α* and *β*, type III); SC express only NRG1 type I/II isoforms (Figure 2). The glial gene GFAP is expressed by SC, OEC, and RT4-D6P2T; S100 and p75 are expressed by all cell types, mostly by SC and RT4-D6P2T (Figure 3).

**Figure 4:**
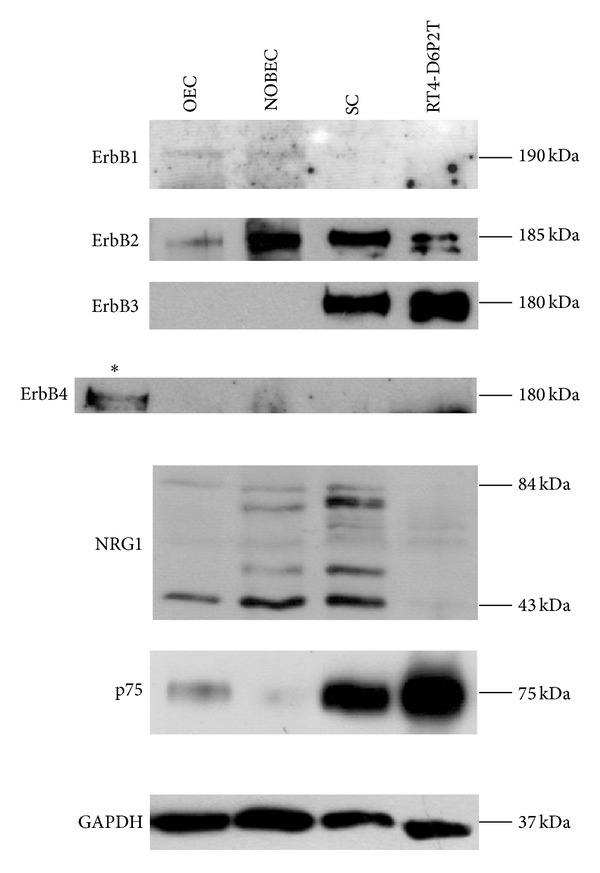
Western blot analysis confirms that primary cultures and cell lines derived from OEC and SC express different levels of ErbB receptors and glial proteins. Western blot analysis of proteins extracted from OEC, NOBEC, RT4-D6P2T, and SC were analyzed with antibodies directed to ErbB1, ErbB2, ErbB3, ErbB4, NRG1, p75, and GAPDH. Different NRG1 isoforms are expressed by OEC, NOBEC, and SC. An asterisk indicates a positive control for ErbB4 expression (a cell line stably expressing ErbB4 [18]).

Immunoblot analysis was performed to confirm protein expression of some of the genes analyzed by qRT-PCR (Figure 4). A faint band corresponding to ErbB1 protein is visible in OEC and NOBEC samples; ErbB2 is expressed, at different levels, in all samples; ErbB3 is strongly expressed by SC and RT4-D6P2T and lowly expressed by NOBEC; ErbB4 is not detectable and for this reason a positive control was added in the Western Blot.

Different NRG1 isoforms are detectable in OEC, NOBEC, and SC. RT4-D6P2T cell line does not express NRG1.

### 3.2. Ecto-ErbB4 Stimulates NOBEC Migration

It has been shown that SC migrate following stimulation with soluble NRG1 and that the removal of soluble NRG1 with recombinant soluble receptors, such as the soluble extracellular fragment of ErbB3, strongly reduces SC migration [26].

NOBEC are characterized by high migratory activity [27], which could be due to an autocrine stimulation mediated by endogenous soluble NRG1 on endogenous ErbB3-ErbB2 heterodimer.

**Figure 5:**
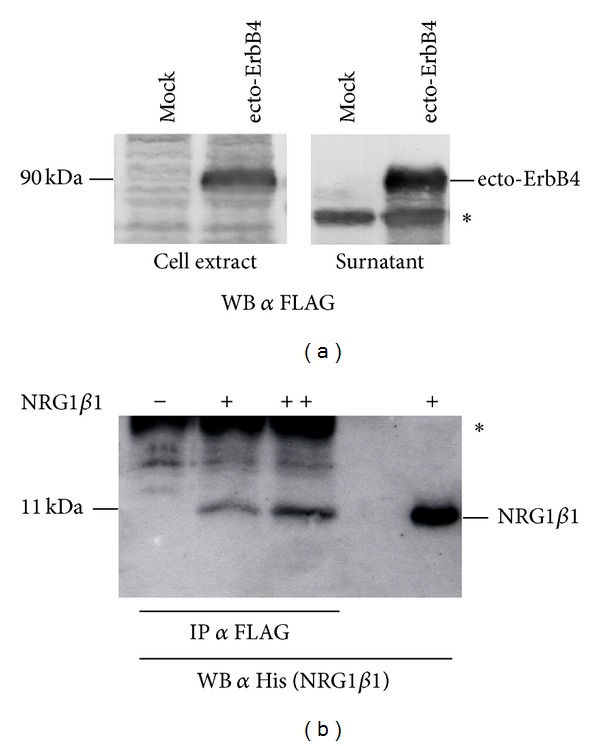
Recombinant ecto-ErbB4-FLAG is expressed and released by cells and is able to interact with soluble NRG1. (a) The correct expression of ecto-ErbB4-FLAG was assayed both in the cell extract and in the conditioned medium (surnatant) of transiently transfected COS7 cells (ecto-ErbB4). Mock samples are COS7 cells transfected with the empty vector. The asterisk indicates an unspecific band. (b) Different amounts of soluble recombinant NRG1*β*1 tagged with 6 histidine were incubated with conditioned medium of COS7 cells transiently expressing recombinant ecto-ErbB4-FLAG. A FLAG coimmunoprecipitation was performed, followed by Western blot against histidine to recognize NRG1*β*1. The asterisk indicates the band corresponding to the primary antibody used for coimmunoprecipitation.

To verify this hypothesis, a construct to express the soluble extracellular domain of ErbB4 (ecto-ErbB4) tagged with a FLAG was produced (see Materials and Methods). By Western blot analysis the expression of ecto-ErbB4-FLAG was assayed both in the cell extract and in the supernatant of transiently transfected COS7 cells, to verify that the soluble extracellular domain of ErbB4 was expressed and released in the extracellular environment (Figure 5(a)).

A coimmunoprecipitation assay was performed to verify the ability of ecto-ErbB4-FLAG to interact with soluble NRG1: different amounts of soluble recombinant NRG1*β*1 tagged with 6 histidine [13] were incubated with conditioned medium containing recombinant ecto-ErbB4 tagged with FLAG. A coimmunoprecipitation against FLAG (ecto-ErbB4) was performed and coimmunoprecipitated proteins were analyzed by Western blot using an antibody against histidine (NRG1*β*1). Data confirm that ecto-ErbB4 and NRG1*β*1 interact (Figure 5(b)).

**Figure 6:**
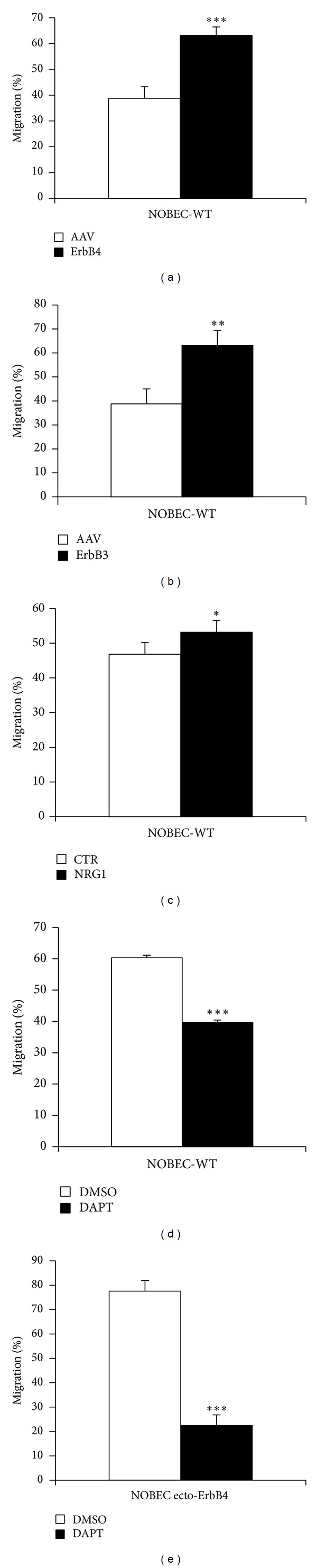
NOBEC transiently expressing ecto-ErbB4 and ecto-ErbB3 migrate more than control cells and their migration is inhibited by DAPT treatment. Migration activity of NOBEC transiently transfected with empty vector (AAV) was compared with migration activity of NOBEC expressing ecto-ErbB4 (ErbB4, Panel (a)) or ecto-ErbB3 (ErbB3, Panel (b)). Soluble recombinant NRG1*β*1 stimulates NOBEC wild type (WT) migration (Panel (c)). NOBEC wild type (Panel (d)) or NOBEC expressing ecto-ErbB4 (Panel (e)) were pretreated for three days with 100 *μ*M DAPT (*γ*-secretase inhibitor) or DMSO (mock control). Cell migration was assessed by Transwell assays. Values represent the average of three biological replicates performed as technical triplicates. Values of each replicate are expressed in percentage with respect to the total number of cells that migrated in that experiment (∗∗, *P* ≤ 0.01; ∗∗∗, *P* ≤ 0.001).

A three-dimensional migration assay (transwell assay) was performed using NOBEC transiently transfected with the expression vector for ecto-ErbB4-FLAG (Figure 6(a)). Contrary to what expected, Transwell assays showed that the expression of the soluble extracellular portion of the receptor ErbB4 increased cell migration. The same results were obtained when NOBEC were transiently transfected with an expression vector for ecto-ErbB3-FLAG (Figure 6(b)).

### 3.3. Ecto-ErbB4-NRG1 Interaction

The promigratory activity elicited by ecto-ErbB4 could be explained by two different models.

1. ecto-ErbB4, sequestering endogenous soluble NRG1, stimulates migration. This hypothesis would be confirmed if soluble NRG1 would have an inhibitory effect on NOBEC migration. To test this hypothesis a Transwell assay was performed in the presence of 50 ng/mL soluble recombinant NRG1*β*1. Transwell assay analysis demonstrates that NRG1*β*1 does not inhibit migration and, indeed, it slightly stimulates migration (Figure 6(c)). Therefore, the first model does not explain the ecto-ErbB4 mediated migration.
2. ecto-ErbB4, interacting with transmembrane NRG1, stimulates migration. Actually, it is known that transmembrane NRG1 type III isoforms are able to mediate reverse signaling [9, 10]: upon interaction with ErbB receptor, transmembrane NRG1 undergoes a proteolytic cleavage mediated by a *γ*-secretase, which releases a cytoplasmic fragment which is able to translocate into the nucleus, mediating reverse signaling.

To verify the expression of NRG1 type III isoforms, an RT-PCR was carried out using specific primers that amplify these isoforms on RNA extracted by NOBEC wild type and NOBEC expressing ecto-ErbB4. RT-PCR confirmed that these NRG1 isoforms are expressed by NOBEC (data not shown), both wild type and expressing ecto-ErbB4.

To validate the reverse-signaling hypothesis, a Transwell migration assay was performed by treating NOBEC expressing ecto-ErbB4 and wild type (WT) NOBEC with DAPT, a specific inhibitor of *γ*-secretase, the enzyme which mediates NRG1 proteolytic cleavage, releasing a cytoplasmic fragment.

In both cell lines, treatment with DAPT inhibits the migration in a consistent way. However, the migration is inhibited more strongly in NOBEC expressing ecto-ErbB4, supporting the hypothesis that this soluble fragment, binding to NRG1-typeIII, leads to the production of a cytoplasmic fragment responsible of the stimulus for migration. The migration is inhibited by 75% in NOBEC expressing ecto-ErbB4 (Figure 6(e)), while only by 25% in wild type NOBEC (Figure 6(d)). Actually, there are numerous signaling pathways that require the presence of *γ*-secretase for the propagation of the signal; therefore, treatment with DAPT could interfere also with the signaling produced by other signal transduction pathways.

### 3.4. NRG1 Cytoplasmic Fragment Stimulates NOBEC Migration

**Figure 7:**
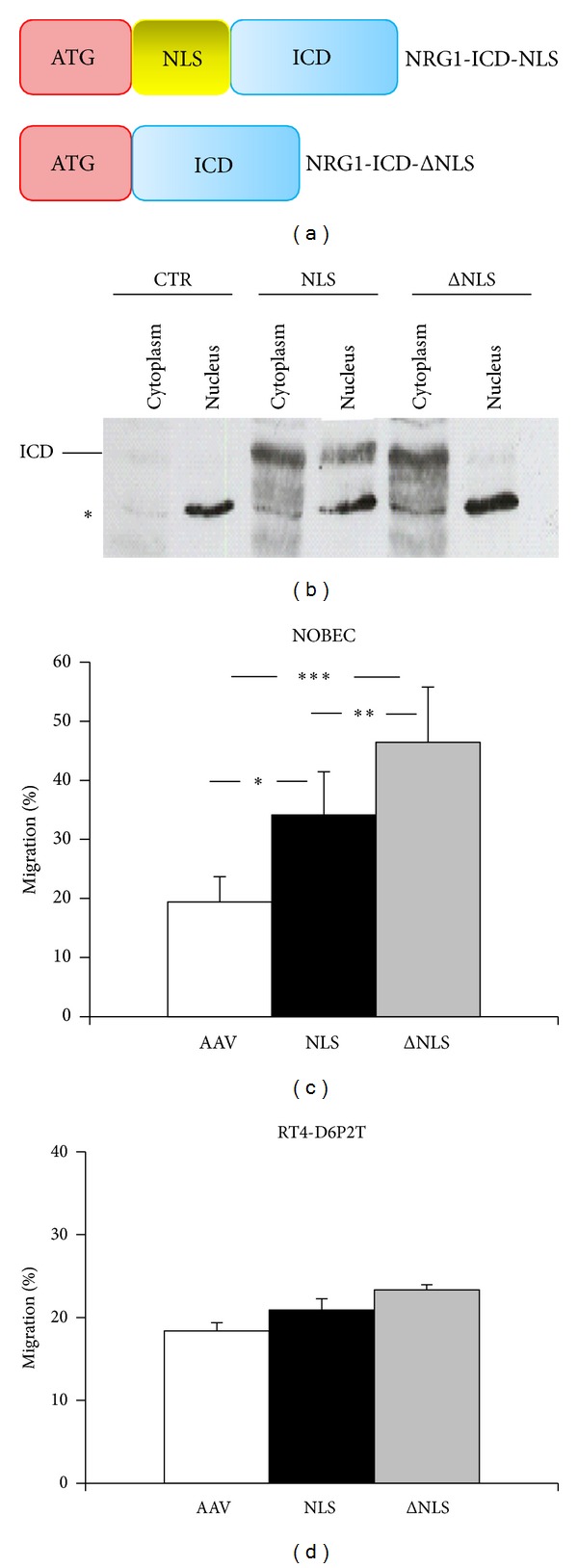
The expression of NRG1 intracellular domain stimulates cell migration. (a) To express the NRG1 intracellular domain (ICD), two constructs were prepared: one containing the nuclear localization sequence (NLS) and one lacking it (ΔNLS). ICD is shown in blue, NLS in yellow, and the inserted ATG in red. (b) Validation of nuclear and cytoplasmic localization of NRG1-ICD fragments. Nuclear and cytoplasmic proteins were extracted from mock (CTR) and NRG1 transfected COS7 cells and subjected to SDS-PAGE and Western blot analysis. Membranes were incubated with anti-NRG1 (sc-348) antibody. Asterisk indicates an unspecific band. (c) NOBEC transiently expressing the NRG1 intracellular domain (ICD), containing (NLS) or lacking (ΔNLS) the nuclear localization sequence, were assayed for migration activity; data show that the cytoplasmic protein confers a migratory activity higher than the migratory activity conferred by the nuclear protein. (d) RT4-D6P2T cells transiently expressing the NRG1 intracellular domain (ICD), containing (NLS) or lacking (ΔNLS) the nuclear localization sequence, were assayed for migration activity. No statistical difference between cells transfected with the empty vector and cells transfected with the two constructs was observed. Values represent the average of three biological replicates performed as technical triplicates. Values of each replicate are expressed in percentage with respect to the total number of cells that migrated in that experiment (∗, *P* ≤ 0.01; ∗∗, *P* ≤ 0.01; ∗∗∗, *P* ≤ 0.001).

To investigate the role of the cytoplasmic fragment of NRG1 produced after interaction with ErbB receptors, two different constructs were prepared to express the NRG1 intracellular domain (ICD): NRG1-ICD-NLS, containing the* nuclear localization sequence* (NLS), and NRG1-ICD-ΔNLS, missing NLS (Figure 7(a)). NLS is a sequence consisting of eight amino acids (KTKKQRKK) following the transmembrane portion of the protein, present in all NRG1 isoforms containing an ICD [9].

Specific primers were designed with the insertion of an artificial ATG to allow the expression of the protein in transfected cells; in one primer the nuclear localization sequence was included; in the other one it was omitted. To verify that the nuclear localization sequence (NLS) inserted into the construct NRG1-ICD-NLS works properly, a Western blot analysis of nuclear and cytoplasmic proteins was performed (Figure 7(b)).

Western blot data show that cells transfected with the construct NRG1-ICD-NLS express the protein both in the cytoplasm and in the nucleus, while cells transfected with the construct NRG1-ICD-ΔNLS, lacking the nuclear localization sequence, express the protein predominantly in the cytoplasm. A barely detectable signal can be observed also in the nucleus; this could be due to the fact that this fragment interacts with proteins, such as LIMK1 [28], able to enter the nucleus carrying associated proteins.

Transwell migration assays were performed with wild type NOBEC transiently transfected with NRG1-ICD-NLS and NRG1-ICD-ΔNLS (Figure 7(c)).

Migration assay analysis shows that transient expression of the NRG1 cytoplasmic domain able to translocate into the nucleus confers a migratory activity that is significantly higher compared to control cells. Unexpectedly, the expression of the NRG1 cytoplasmic fragment lacking NLS confers to the cells a migratory activity that is significantly higher than control and of the cells expressing the isoform with NLS. These data suggest that NRG1-ICD plays a role in the stimulation of the migratory activity, when it is in the cytoplasm.

### 3.5. NRG1 Cytoplasmic Fragment Does Not Stimulate RT4-D6P2T Migration

To understand if the cytoplasmic fragment of NRG1 type III is able to confer migratory activity to cells that do not express endogenously any isoform of NRG1, the cell line RT4-D6P2T was transiently transfected with vectors to express NRG1-ICD-NLS and NRG1-ICD-ΔNLS and migratory activity was analyzed by Transwell assays (Figure 7(d)).

Data show that transient expression of NRG1-ICD does not confer migration activity, suggesting that these cells lack pivotal factors necessary to mediate NRG1 migratory signal transduction.

### 3.6. Signal Transduction Pathways Activated by Ecto-ErbB4 on the NOBEC Cell Line

It has been shown that in neurons, expressing transmembrane NRG1-typeIII, stimulation with soluble ErbB4 fragment produces an increase in AKT phosphorylation, while ERK phosphorylation remains unchanged [29].

To further investigate the reverse signaling mediated by ecto-ErbB4 in NOBEC cells, a time course of stimulation was performed.

**Figure 8:**
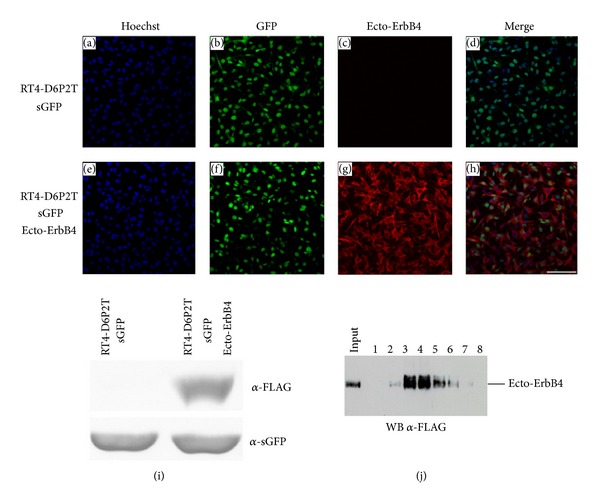
Following lentivirus (LV) infection, RT4-D6P2T successfully express GFP and ecto-ErbB4-FLAG. Confluent cells infected with* LV-sGFP* only (Panels (a)–(d)) or with* LV-sGFP* and* LV-ecto-ErbB4-FLAG* (Panels (e)–(h)) were stained with anti-GFP (green) and anti-FLAG (red) antibody. Nuclei were stained with Hoechst (blue). Scale bar 100 *μ*m. Panel (i)-Western blot analysis of RT4-D6P2T infected with LV-sGFP only, or with LV-sGFP and LV-ecto-ErbB4-FLAG. (j) Recombinant ecto-ErbB4-FLAG peptide was purified from RT4-D6P2T conditioned medium using ANTI-FLAG affinity gel and eluted using FLAG peptide. Eluted fractions were analyzed by Western blot to identify the positive fractions to be frozen and used for the following experiments.

**Figure 9:**
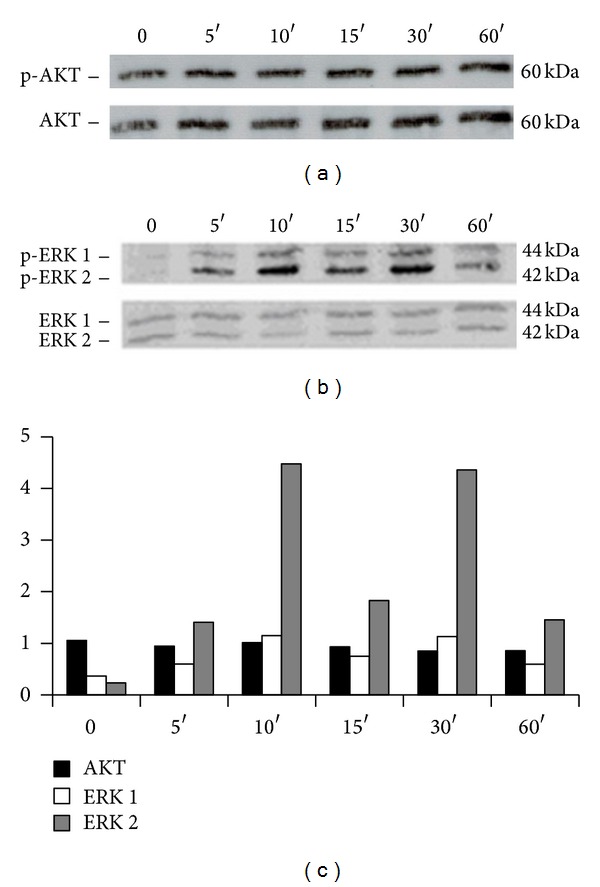
Ecto-ErbB4 stimulates ERK phosphorylation in NOBEC. Western blot analysis of NOBEC cells stimulated with recombinant soluble ecto-ErbB4-FLAG for 0, 5, 10, 15, 30, and 60 min. Western blot was analyzed with antibodies anti-p-AKT and AKT (Panel (a)) and anti-p-ERK 1/2 and ERK (Panel (b)). (c) Bands were analyzed by quantifying the intensity of the pixels per mm^2^ (Image J). The values of the bands corresponding to phosphorylated proteins were normalized to the intensity of the bands corresponding to the total proteins.

To obtain pure and highly concentrated recombinant protein to stimulate cells, RT4-D6P2T were transduced with a lentivirus expressing ecto-ErbB4-FLAG (Figure 8). Conditioned medium was collected from RT4-D6P2T-ecto-ErbB4-FLAG cells to purify ecto-ErbB4-FLAG through a chromatographic column (Figure 8(j)). NOBEC cells were starved 24 h in serum-free medium and then stimulated with the purified recombinant protein for 5, 10, 15, 30, and 60 min. By Western blot analysis AKT and ERK 1/2 phosphorylation was analyzed (Figure 9). Data analysis shows that in this glial cell model, contrary to neuron cells, AKT phosphorylation does not change, while ERK phosphorylation changes. In particular, there is a strong increase in ERK2 phosphorylation and there is a low increase in ERK1 phosphorylation (Figure 9(c)).

## 4. Discussion

The study of peripheral nerve repair and regeneration is an emerging issue in biomedicine since, although peripheral nerves retain a significant capacity for spontaneous regeneration after lesion, regeneration is almost never complete [30] and functional recovery is often unsatisfactory [31].

Innovative strategies to improve peripheral nerve regeneration are expected to arise from the manipulation of the several trophic factor loops playing a role in this process. Among them the neuregulin1/ErbB system is attracting much attention because of its action on SC [3, 4]. The aim of this study was to further explore the role of the neuregulin1/ErbB system and the potential of its manipulation.

### 4.1. Neuregulin1 Isoforms and ErbB Receptors Are Differentially Expressed in SC and OEC (Both Primary Cultures and Cell Lines)

The NRG1/ErbB system is involved in the development of the central and peripheral nervous system, in which different members of the ErbB receptor family and different NRG1 isoforms control processes are opposed to each other, such as proliferation and cell death [32]. NRG1/ErbB signaling plays a fundamental role in SC precursor growth and in interactions between SC (expressing ErbB2-ErbB3 and soluble type I/II NRG1) and axons (mainly expressing transmembrane NRG1-type III). NRG1-type III plays an instructive role on myelination and SC development, determining the ensheathment fate of axons: its reduced expression causes hypomyelination [33]. Moreover, NRG1-type III absence [34] or the lack of coreceptors ErbB3 [35] or ErbB2 [36, 37] gives rise to mice without or with severely reduced amount of SC precursors.

In this paper, primary cultures of SC and OEC and immortalized cultures of SC (RT4-D6P2T) and OEC (NOBEC), commonly used in* in vitro* analysis, were compared and characterized in terms of ErbB receptors, NRG1 isoforms, P75, S100, and GFAP expression.

Data presented here have shown that RT4-D6P2T cells are very similar to primary SC, since they express mRNAs for ErbB2, ErbB3, and the glial genes GFAP, P75, S100, and do not express mRNA for ErbB1 and NRG1 type III. RT4-D6P2T do not, however, show detectable expression of NRG1, while SC express NRG1 type I/II. OEC and NOBEC cell lines express all NRG1 isoforms; ErbB1 and ErbB2 are expressed in both OEC and NOBEC, while ErbB3 is slightly expressed. ErbB4 mRNA is barely expressed in OEC and SC, and its protein is not detectable.

### 4.2. Ecto-ErbB4* In Vitro* Expression Increases NOBEC Migration

Following the characterization of the four cell populations as well as the immunohistochemical assay to compare OEC and NOBEC, we decided to proceed with the study of NRG1/ErbB system manipulation using the NOBEC line, which expresses the transcripts for different NRG1 isoforms.

Data previously obtained in our laboratory showed that NOBEC have a high migratory activity [27]. We speculated whether the high migration was due to an autocrine loop induced by self-produced NRG1; in fact, it is known that glial cells migrate following stimulation with NRG1 [26]. To negatively interfere with NOBEC migration, we planned to subtract soluble NRG1 using the recombinant soluble extracellular fragment of ErbB3 or ErbB4 (ecto-ErbB). Contrary to what expected, results showed that ecto-ErbB3 and ecto-ErbB4 expression significantly increases cellular migration.

We hypothesized that this migration increase was due to the interaction between the soluble portion of ErbB4 receptor and the NRG1 type III expressed by the NOBEC cell line, through a process of back-signaling that this transmembrane isoform of NRG1 is able to generate. Usually, this NRG1 isoform is expressed by axons in which the back-signaling is mediated by the action of a *γ*-secretase that, following ligand-receptor interaction, causes the release of a NRG1 cytoplasmic fragment able to translocate into the nucleus [9], where it can regulate transcription of genes involved in neuronal development [38].

Actually, we demonstrated that NRG1 intracellular domain (NRG1-ICD) stimulates migration. Particularly, we saw that the fragment localized in the cytoplasm, more than the fragment localized in the nucleus, plays an important role in the stimulation of the migratory activity. This action could be due to the interaction between this intracellular fragment and cytoplasmic binding partners, such as the LIM kinase1/LIMK1 [28] that is able to shuttle between the cytoplasm and the nucleus, by regulating gene transcription through interaction with the actin cytoskeleton [39]. Interaction between NRG1-ICD and LIMK1 has been found at the level of synapses, where LIMK1 is involved in the regulation of the reorganization of actin filaments in the neuritic protuberances [28, 40, 41].

Transfection of NRG1-ICD in cells that do not express any isoform of NRG1, like RT4-D6P2T cells line, does not affect migration, thus suggesting that these cells lack elements necessary for the signal transduction mediated by NRG1.

Moreover, we found that stimulation of NOBEC with the soluble portion of ErbB4 increases ERK phosphorylation without affecting AKT phosphorylation, contrary to what happens in neuron cells, in which there is an increase of AKT phosphorylation [29]; these results suggest that the back-signaling mediated by NRG1 type III activates signal transduction pathways which differ according to the cell type.

### 4.3. Conclusion

This study shows that stable cell lines and the corresponding primary cultures have many characteristics in common, thus suggesting that cell lines are a good model for* in vitro* studies. On the other hand, these results show that glial olfactory ensheathing cells and Schwann cells differ for the expression of some proteins, thus suggesting that the choice of the cell model for* in vitro *studiesshould be done carefully, after investigating the expression of the genes of interest.

Finally, these results suggest that recombinant ecto-ErbB4 can be used not only to sequester soluble NRG1, but also to activate transmembrane NRG1 through a back signaling pathway that can stimulate cell migration. Thus, ecto-ErbB4, a protein fragment endogenously released by cells expressing the cleavable isoform of the NRG1 receptor ErbB4 [42], turns out to be a potential tool to manipulate* in vivo* the neuregulin1/ErbB system. Nevertheless, further studies are required to design a strategy for a finely tuned ecto-ErbB4 delivery, to investigate the possibility to promote posttraumatic peripheral nerve regeneration.

## Figures and Tables

**Table 1:** Primers used for quantitative real time-PCR reaction.

GENE	Forward primer (5′-3′)	Revrse primer (3′-5′)	Sequence accession number	Amplicon
ErbB1	CACCACGTACCAGATGGATG	CGTAGTTTCTGGGGCATTTC	U52529	85 bp
ErbB2	CACTTGGAGCTTACCTACGTGCC	ACCTGGTTGTGAGCGATGAGC	NM_017003	98 bp
ErbB3	TGCTGACCTTTCCTTCCTGCAATGG	GAGGTTAGGCAAGGGAAGCACAGAG	NM_017218.2	94 bp
NRG1 type I	GGCGCAAACACTTCTTCATCCAC	AAGTTTTCTCCTTCTCCGCGCAC	AF 194993	82 bp
NRG1 type III	CCCTGAGGTGAGAACACCCAAGTC	TGGTCCCAGTCGTGGATGTTGATG	AF 194439	103 bp
NRG1 α	GCGGAGAAGGAGAAAACTTTC	TTGCTCCAGTGAATCCAGGTTG	RNU-02323	112 bp
NRG1 β	GCGGAGAAGGAGAAAACTTTC	AACGATCACCAGTAAACTCATTTGG	RNU-02322	116 bp
GFAP	GAGGCAGTGGCCACCAGTAACATG	GGAAGCAACGTCTGTGAGGTCTGC	NM_017009	81 bp
S100	GGGTGACAAGCACAAGCTGAAGAA	TTGTCCACCACTTCCTGCTCTTTG	NM_013191	102 bp
P75	AGCAGACCCATACGCAGACTG	TCTCTACCTCCTCACGCTTGG	NM_012610	95 bp
